# Data quality and factor analysis of the Danish version of the Relationship Scale Questionnaire

**DOI:** 10.1371/journal.pone.0176810

**Published:** 2017-05-04

**Authors:** Christina Maar Andersen, Anette Fischer Pedersen, Anders Helles Carlsen, Frede Olesen, Peter Vedsted

**Affiliations:** 1 Department of Psychology, University of Southern Denmark, Odense, Denmark; 2 Research Centre for Cancer Diagnosis in Primary Care (CaP), Research Unit for General Practice, Department of Public Health, Aarhus University, Aarhus, Denmark; Universitat Wien, AUSTRIA

## Abstract

**Background:**

The Relationship Scale Questionnaire (RSQ) is a widely-used measure of adult attachment, but whether the results obtained by the RSQ fit the attachment construct has only been examined to a limited extent.

**Objective:**

The objectives of this study were to investigate the psychometric properties of the Danish translation of the RSQ and to test whether the results are consistent with the hypothesized model of attachment.

**Methods:**

The study included two samples: 602 general practitioners and 611 cancer patients. The two samples were analyzed separately. Data quality was assessed by mean, median and missing values for each item, floor and ceiling effects, average inter-item correlations and Cronbach’s α for each subscale. Test-retest was assessed by intra-class correlations among 76 general practitioners. A confirmatory factor analysis was conducted to establish evidence of the four proposed subscales. Due to an inadequate fit of the model, data was randomly split into two equally sized subsamples and an exploratory factor analysis was conducted for all 30 items in the first subsample comprised of 286 cancer patients and 285 general practitioners. The EFA yielded a three-factor structure which was validated through a confirmatory factor analyses in a second subsample comprised of 278 cancer patients and 289 general practitioners.

**Results:**

The data quality of the RSQ was generally good, except low internal consistency and low to moderate test-retest reliability. The four subscales of the RSQ were not confirmed by the confirmatory factor analysis. An exploratory factor analysis suggested a three-factor solution for both general practitioners and patients, which accounted for 61.1% of the variance among general practitioners and 62.5% among patients. The new three-factor solution was verified in a confirmatory factor analyses.

**Conclusion:**

The proposed four-factor model of the RSQ could not be confirmed in this study. Similar challenges have been found by other studies validating the RSQ. An alternative three-factor structure was found for the RSQ.

## Introduction

During decades, attachment-related research has been extended to the field of health psychology. Studies have investigated adult attachment in the areas of chronic pain [1–3], bipolar disorder [4], alcohol addiction [5], chronic disease [6], chronic whiplash disorder [7], diabetes [8,9], troublesome emergency department patients [10], general practitioner interventions for medically unexplained symptoms [11], and primary healthcare utilization [12]. Attachment theory presents a model for understanding illness behavior as it describes how early interactions between caregiver and child create lifelong patterns of stress response, receptivity to social support, and vulnerability to illness [13]. Self-report questionnaires are most often used for research in adult attachment. This calls for more knowledge on the psychometric properties and the underlying factor structure of the applied measures.

### Attachment theory

According to Bowlby, founder of the attachment theory, attachment is the emotional bond that is created from interactions between a child and its caregiver. If the caregiver is responsive and sensitive, the caregiver will be a safe base from which the child can explore the world and return to when feeling scared [14,15]. The quality of the caregiving refers to the caregiver’s sensitivity toward the child’s needs and will affect the child’s sense of self, the child’s regulation of emotions, and how the child will enter relationships with other people throughout life [14,16].

### Measuring adult attachment

Hazan and Shaver were the first to measure attachment styles in adults. Their scale was based on Ainsworth’s three childhood attachment styles: secure, insecure avoidant, and insecure ambivalent [17]. Later, Bartholomew argued that avoidance in adult intimacy should be categorized into two different styles depending upon the person’s model of self [18]. Her model was based on Bowlby’s concept of internal working models, in which individual differences in adult attachment can be systematized in terms of the intersection of two dimensions (*model of self* and *model of other*). Each of the two dimensions can be dichotomized as positive or negative, which in combination form four attachment subscales (*Secure*, *Preoccupied*, *Dismissing*, and *Fearful*). Individuals with a secure attachment style have a positive model of self and other. Preoccupied individuals have a negative model of self, but a positive model of other. Individuals with a dismissing-avoidant attachment style have a positive model of self, but a negative model of other, and fearful-avoidant individuals have a negative model of self and other [19].

Numerous other self-report measures assessing attachment among adults have also been developed [20]. Most of them measure attachment in romantic relationships, whereas the Relationship Scale Questionnaire (RSQ) measures the individual’s general approach to close relationships. The RSQ is widely used [20–28], but whether the results obtained by the RSQ fit the attachment construct has only been examined to a limited extent. Studies trying to validate the four subscales in the original RSQ and a Spanish version of the RSQ have not been successful so far [29,30]. Thus, the present study investigates the psychometric properties of the Danish translation of the RSQ and tests whether the results are consistent with the hypothesized model of attachment. The validity of the RSQ is tested in two different samples i.e. cancer patients and general practitioners. Cancer patients’ attachment system is expected to be activated due to their illness. The RSQ has not to our knowledge been validated in a sample of individuals where the likelihood of the attachment system being activated was high. The attachment system of the general practitioners is not expected to be activated to the same degree and will thus serve as a comparison in the validation.

## Methods

### Ethical issues

Participants for this study were recruited through two different studies. These studies were approved by the Danish Data Protection Agency (J.no. 2011-41-6632 and J.no. 2011-41-6609) and the Danish Health and Medicines Authority. According to Danish law, approval by the National Committee on Health Research Ethics was not required as no biomedical intervention was performed and no biological material was collected.

### Participants

Participants for this study comprised of two different samples. The first sample consisted of 602 general practitioners from the Central Denmark Region who participated in a large questionnaire survey on general practitioners’ mental health and psychosocial work environment [31] with a response rate of 72.1%. The test-retest reliability was examined by asking 76 of the general practitioners to fill out the RSQ on two different occasions in a period ranging from 9 to 20 months between the first and the second time. The long period until retest was chosen in consideration of the stability of attachment styles.

The second sample consisted of 611 incident cancer patients from the Central Denmark Region who participated in a questionnaire survey on diagnostic intervals during cancer diagnosis. The 611 patients were a random consecutive sample identified through the Danish National Patient Registry [32]. In the projects on diagnostic intervals during cancer diagnosis a total of 4,509 patients were invited to participate with a response rate of 67%. The 611 patients in this study were the first returned questionnaire from that survey.

### Measurement techniques

The RSQ consists of 30 items measured on a 5-point range scale ranging from 1 (*not at all like me*) to 5 (*very much like me*). Items 6, 9, and 28 are reverse-scored. According to Griffin & Bartholomew, the four attachment styles (i.e. Secure, Preoccupied, Dismissing and Fearful) can be derived by computing the mean rating of the items for each subscale [33]. The model of self and the model of other can then be derived by the following equations:

Model of self: (Secure + Dismissing) − (Fearful + Preoccupied)

Model of other: (Secure + Preoccupied) − (Fearful + Dismissing)

The RSQ is based on items from Hazan and Shaver's three-category attachment styles, Bartholomew and Horowitz's four-category attachment styles, and Collins and Read's attachment measure [33]. Only 17 items of the RSQ are used to produce the four attachment styles. Item 6 appear in the scoring as both reverse-scored and direct-scored [33]. This leaves 13 items redundant in the original scoring, however, these items can be used for deriving other attachment styles or dimensions. See Kurdek’s paper for an overview [34]. It is unclear from the scoring manual of the RSQ whether only the 17 items can be used on their own or whether all 30 items have to be included when using the RSQ for accessing people’s attachment style. Being presented with all 30 items may give a different response from participants than only being presented with the 17 items.

### Translation and cultural adaptation

The RSQ was translated and adapted from English into Danish on the basis of WHO guidelines [35]. The translation process included a forward-backward translation, expert panel discussion, pilot test, and cognitive interviews. The forward translation was carried out by two psychologists and one language expert in Danish and English with Danish as native language. The backward translation was carried out by a native English language expert. The expert panel comprised of the two language experts and the two psychologists. The expert panel discussions resulted in minor adaptations of the Danish version of the RSQ, mainly to clarify cross-cultural language differences. An example of a cultural difference was the word *romantic partners*, which cannot be translated directly into an unambiguous Danish concept. Instead, we chose to use the term *partners* (in Danish: *partnere*) and to add a bracket with the words *current and previous* (in Danish: *nuværende og tidligere*) to explain the plural of the word *partners* and yet prevent that people would be offended by an indication of promiscuity.

Cognitive semi-structured interviews were conducted with six general practitioners and six cancer patients to determine whether the Danish version of the RSQ was comprehensible and meaningful. The focus of the interviews was also to establish whether the response categories of the scale were sufficient and whether important concepts were understood correctly. Minor adjustments were made on the basis of the interviews.

The RSQ was pilot-tested among 30 general practitioners from the North Denmark Region. The pilot testing did not give rise to further alterations of the RSQ. A report on the translation process can be requested from the first author.

### Statistical analysis

The analyses consisted of four parts. First, we assessed the data quality of the items and the four subscales. Second, we conducted a confirmatory factor analysis (CFA) to validate the four-factor structure proposed by Bartholomew and Horowitz [19]. Third, as the proposed four-factor structure could not be established, each sample was randomly split into two equally sized subsamples and an exploratory factor analysis (EFA) was conducted to investigate alternative factor structures among all the 30 items in each of the two subsamples (cancer patient subsample and general practitioner subsample). Fourth, the alternative three-factor structure established by the EFA was validated using a CFA in each of the other two subsamples. The data of the general practitioners and the patients were assessed separately as the two samples differed on important aspects such as age, health status, and socioeconomic position.

Data quality was assessed in terms of mean with standard deviation, median, and percentage of missing data for each of the 30 items. Floor and ceiling effects were defined as lowest or highest possible sum scores of more than 15% of the respondents, respectively [36,37]. To examine the subscales’ internal consistency, Cronbach’s α and the average inter-item correlation were calculated. A measure of acceptable internal consistency is generally considered to be between 0.70 and 0.90 for Cronbach’s α [38]. In contrast to Cronbach’s α, the average inter-item correlation is independent of the number of items and sample size. An average inter-item correlation of at least 0.50 is generally considered satisfactory [39]. Test-retest reliability was assessed by interclass correlation (ICC) [40] for the four subscales. An ICC value of 0.70 is generally considered acceptable [40].

The underlying factor structure was examined for the 17 items of the RSQ that constitute the four subscales. In the CFA, we allowed inter-correlation among the four latent factors. In the EFA, we used the eigenvalue-one criterion and oblique factor rotation to allow for correlation among the latent factors [41] and the weighted least squares means and variance adjusted (WLSMV) estimator for categorical indicators and list-wise deletion of missing values.

To assess the goodness of fit for the CFA and the EFA, the following five indices were used [41,42]:

1. Chi-squared goodness-of-fit statistics (χ^2^) measures the overall model fit and the magnitude of discrepancy between the sample and the fitted covariance matrices; a significance test with *p* values > 0.01 indicates a good fit.
2. Comparative fit index (CFI) compares the sample covariance matrix with a null model; values range between 0.0 and 1.0, and values close to 1.0 indicate very good fit.
3. Tucker-Lewis Index (TLI) adjusts for number of model parameters and is interpreted in the same way as CFI.
4. The root mean square error of approximation (RMSEA) expresses the lack of fit per degree of freedom of the model and accounts for sample size; values ≤ 0.05 indicate very good fit, values > 0.05–0.08 indicate good fit, and values ≥ 0.10 indicate poor fit.
5. Standardized root mean square residual (SRMR) is the average of the differences between the observed and predicted correlations and ranges from 0 to 1; values < 0.08 indicate good fit.

Data were analysed using STATA, version 13 [43], and Mplus, version 7.11 [44].

## Results

### Description of the study populations

A total of 602 general practitioners (316 men and 286 women) filled out the RSQ; their average age was 52.1 years (SD = 8.6). A total of 611 cancer patients (312 men and 299 women) filled out the RSQ; their average age was 64.4 years (SD = 12.4).

### Data quality

The number of general practitioners who answered each item ranged from 586 to 595, with missing values ranging from 1.2–2.7% (Table 1). The number of patients who answered each item ranged from 578 to 604, with missing values ranging from 1.2–5.4% (Table 1). No floor or ceiling effect was found in the four subscales for GPs or patients.

**Table 1:** Data quality, mean, SD, median, item response, and missing values for the 17 items in the RSQ questionnaire on attachment; Results for both general practitioners and cancer patients.

Items	General Practitioners					Cancer Patients				
	N	Mean	SD	Median	Missing (%)	N	Mean	SD	Median	Missing (%)
1. I find it difficult to depend on other people.	590	2.59	1.14	2	2.0	604	3.30	1.34	3	1.2
2. It is very important to me to feel independent.	587	3.13	1.09	3	2.5	601	3.72	1.19	4	1.6
3. I find it easy to get emotionally close to others.	590	3.72	0.96	4	2.0	600	3.29	1.20	3	1.8
4. I want to merge completely with another person.	589	2.97	1.05	3	2.2	600	2.87	1.24	3	1.8
5. I worry that I will be hurt if I allow myself to become too close to others.	589	1.91	0.87	2	2.2	598	2.56	1.21	3	2.1
6. I am comfortable without close emotional relationships.	591	1.95	1.00	2	1.8	601	2.67	1.25	3	1.6
7. I am not sure that I can always depend on others to be there when I need them.	588	1.96	1.01	2	2.3	596	2.19	1.27	2	2.5
8. I want to be completely emotionally intimate with others.	588	2.97	1.03	3	2.3	602	2.83	1.21	3	1.5
9. I worry about being alone.	590	2.25	1.09	2	2.0	596	2.91	1.50	3	2.5
10. I am comfortable depending on other people.	588	2.47	1.03	2	2.3	602	2.07	1.22	2	1.5
11. I often worry that romantic partners don’t really love me.	587	1.55	0.82	1	2.5	586	1.61	1.04	1	4.1
12. I find it difficult to trust others completely.	589	1.67	0.83	1	2.2	597	2.19	1.17	2	2.3
13. I worry about others getting too close to me.	589	1.64	0.69	2	2.2	595	2.05	1.10	2	2.6
14. I want emotionally close relationships.	588	3.71	1.08	4	2.3	592	3.16	1.26	3	3.1
15. I am comfortable having other people depend on me.	588	2.91	1.08	3	2.3	597	2.44	1.23	2	2.3
16. I worry that others don’t value me as much as I value them.	588	1.97	0.95	2	2.3	599	2.14	1.17	2	2.0
17. People are never there when you need them.	588	1.49	0.73	1	2.3	592	1.54	0.95	1	3.1
18. My desire to merge completely sometimes scares people away.	587	1.44	0.67	1	2.5	589	1.53	0.86	1	3.6
19. It is very important to me to feel self-sufficient.	588	3.95	0.94	4	2.3	600	4.16	1.05	5	1.8
20. I am nervous when anyone gets too close to me.	587	1.72	0.77	2	2.5	591	1.88	1.04	2	3.3
21. I often worry that romantic partners won’t want to stay with me.	586	1.46	0.73	1	2.7	578	1.50	1.02	1	5.4
22. I prefer not to have other people depend on me.	588	2.34	1.01	2	2.3	594	2.89	1.31	3	2.3
23. I worry about being abandoned.	593	1.68	0.91	1	1.5	587	2.03	1.39	1	3.9
24. I am somewhat uncomfortable being close to others.	595	1.59	0.73	1	1.2	590	1.89	1.07	2	3.4
25. I find that others are reluctant to get as close as I would like.	595	1.72	0.78	2	1.2	590	1.87	1.05	2	3.4
26. I prefer not to depend on others.	595	2.73	1.20	3	1.2	600	3.57	1.34	4	1.8
27. I know that others will be there when I need them.	595	4.00	1.01	4	1.2	599	4.32	1.02	5	2.0
28. I worry about having others not accept me.	595	1.91	0.93	2	1.2	594	1.76	1.11	1	2.8
29. Romantic partners often want me to be closer than I feel comfortable being.	594	1.75	0.90	2	1.3	579	1.83	1.10	1	5.2
30. I find it relatively easy to get close to others.	593	3.48	1.03	3	1.5	596	3.27	1.15	3	2.5

The inter-item correlations for the four subscales ranged from 0.10 to 0.35 for the general practitioners and from 0.19 to 0.46 for the patients. Cronbach’s α ranged from 0.34 to 0.70 for the general practitioners and from 0.40 to 0.68 for the patients for the four subscales (Table 2). The Cronbach’s α for all of the 17 items was 0.72 for the patients and 0.81 for the general practitioners. The average inter-item correlation was 0.19 for both patients and general practitioners. The test-retest reliability (ICC) ranged from 0.56 for the Preoccupied subscale to 0.72 for the Fearful subscale (Table 3).

**Table 2:** Mean, SD, inter-item correlation, Cronbach’s α, and percentage in lowest (floor) and highest (ceiling) score for the four RSQ subscales; Results for both general practitioners and cancer patients.

Subscales	General Practitioners							Patients						
	N	Mean	SD	Inter-item correlation	Cronbach’s α	Floor Score = 1 (%)	Ceiling Score = 5 (%)	N	Mean	SD	Inter-item correlation	Cronbach’s α	Floor Score = 1 (%)	Ceiling Score = 5 (%)
Secure	585	3.39	0.54	0.10	0.34	0.34	0.17	582	3.03	0.60	0.21	0.43	0.69	0.17
Preoccupied	585	2.68	0.53	0.16	0.47	0.17	0.17	584	2.54	0.66	0.19	0.40	2.23	0.17
Dismissing	584	2.83	0.71	0.35	0.70	0.17	0.17	584	3.39	0.82	0.46	0.68	0.51	3.60
Fearful	586	1.94	0.64	0.28	0.68	8.19	0.51	586	2.48	0.76	0.30	0.51	3.75	0.34

**Table 3:** Test-retest reliability of the RSQ measured by interclass correlation (ICC) among 76 general practitioners.

Subscale	N	Sum-score at first test (mean (SD))	Sum-score at retest (mean (SD))	ICC
Secure	76	3.5 (0.5)	3.3 (0.6)	0.63
Preoccupied	76	2.8 (0.6)	2.5 (0.5)	0.56
Dismissing	76	2.8 (0.7)	2.9 (0.6)	0.61
Fearful	75	1.9 (0.7)	2.0 (0.6)	0.72

### Confirmatory factor analysis for the proposed model

All the goodness-of-fit tests for general practitioners and patients indicated poor comparative fit between the present factor structure and the original four-factor structure (Table 4).

**Table 4:** Fit indices for the CFA of the original RSQ factorial structure for general practitioners and patients.

Analyses	Group	N	Chi^2^ (df)	CFI	TLI	RMSEA (95% CI)
CFA	General Practitioners	577	2338.248 (129)	0.610	0.538	0.172 [0.166, 0.178]
	Patients	566	2242.388 (129)	0.465	0.366	0.170 [0.164, 0.176]

*Note*. Chi^2^(df) = Chi-square with degrees of freedom; CFI = comparative fit index; TLI = Tucker-Lewis incremental fit index;

RMSEA(CI) = root mean square error of approximation with 90% CI.

### Exploratory factor analysis

A three-factor solution was suggested for both general practitioners and patients, covering attachment themes similar to those found in other studies [29,30]. These themes were named *Independence*, *Relationship Worry* and *Closeness* and included 14 of the original 30 items. The goodness-of-fit tests were adequately supporting the factorability of the data (Table 5). Item loadings for the three factors are shown in Table 6, which displays all loadings above 0.40. All items were assigned to the same factors for both groups.

**Table 5:** Fit indices for the EFA of the derived factorial structure and the Fit indices of the CFA of these derived factors for general practitioners and patients.

**Fit Indices for the EFA**							
Analyses	Samples	N	Chi^2^ (df)	CFI	TLI	RMSEA (95% CI)	SRMR
EFA	General Practitioners	285	180.751 (52)	0.945	0.904	0.093 [0.079, 0.108]	0.045
	Patients	286	199.549 (52)	0.922	0.863	0.100 [0.085, 0.114]	0.049
**Fit Indices for the CFA of the derived factorial structure**							
Analyses	Samples	N	Chi^2^ (df)	CFI	TLI	RMSEA (95% CI)	SRMR
CFA	General Practitioners	289	239.065 (74)	0.922	0.904	0.088 [0.076, 0.100]	NA
	Patients	278	181.703 (74)	0.927	0.911	0.072 [0.059, 0.086]	NA

*Note*. Chi^2^(df) = Chi-square with degrees of freedom; CFI = comparative fit index; TLI = Tucker-Lewis incremental fit index;

RMSEA(CI) = root mean square error of approximation with 90% CI; SRMR = standardized root mean-square residual; NA = Not Assessed.

**Table 6:** Factors and items with loadings of the derived three factors of the EFA. All Items loaded best for the same factors for both general practitioners and patients.

Table T	General Practitioners			Patients		
	Factor 1	Factor 2	Factor 3	Factor 1	Factor 2	Factor 3
**Factor 1—Independence**						
1. I find it difficult to depend on other people.	0.757	-	-	0.713	-	-
2. It is very important to me to feel independent.	0.781	-	-	0.908	-	-
19. It is very important to me to feel self-sufficient.	0.617	-	-	0.691	-	-
26. I prefer not to depend on others.	0.707	-	-	0.594	-	-
**Factor 2—Relationship Worry**						
11. I often worry that romantic partners don’t really love me.	-	0.724	-	-	0.739	-
16. I worry that others don't value me as much as I value them.	-	0.807	-	-	0.688	-
17. People are never there when you need them.	-	0.573	-	-	0.650	-
23. I worry about being abandoned.	-	0.744	-	-	0.679	-
25. I find that others are reluctant to get as close as I would like.	-	0.709	-	-	0.686	-
28. I worry about having others not accept me.	-	0.711	-	-	0.677	-
**Factor 3—Closeness**						
3. I find it easy to get emotionally close to others.	-	-	0.749	-	-	0.682
4. I want to merge completely with another person.	-	-	0.704	-	-	0.693
8. I want to be completely emotionally intimate with others.	-	-	0.528	-	-	0.714
30. I find it relatively easy to get close to others.	-	-	0.632	-	-	0.676

*Note*. All loadings were significant at 5% level. Only values above 0.4 are displayed, and cells with values beneath 0.4 are marked with a dash (-).

The three factors accounted for 62.5% of the variance among the general practitioners and for 61.1% among the patients. Factor one (General practitioners: eigenvalue = 4.33; Patients: eigenvalue = 3.61) named Independence accounted for 31.0% of the total variance among the general practitioners and for 25.8% among the patients. Factor two (General practitioners: eigenvalue = 2.82; Patients: eigenvalue = 2.92) named Relationship Worry accounted for 20.1% among the general practitioners and for 20.9% among the patients. The third factor (General practitioners: eigenvalue = 1.60; Patients: eigenvalue = 2.01) named Closeness accounted for 11.4% of the total variance among the general practitioners and for 14.4% among the patients.

### Confirmatory factor analysis for the alternative model

The confirmatory factor analyses for the alternative factor structure found in the EFA validated a three-factor solution. All the goodness-of-fit tests for general practitioners and patients indicated an acceptable fit (Table 5). A CFA for the whole sample (general practitioners and patients) showed similar fit statistics (N = 567, Chi^2^ (df) = 329.390 (74), CFI = 0.934, TLI = 0.918, RMSEA (95% CI) = 0.078 (0.087)) as for the individual samples.

For the patients, Cronbach’s α for the derived factors ranged from 0.73 to 0.74 and the inter-item correlation ranged from 0.41 to 0.61. For the general practitioners, Cronbach’s α ranged from 0.77 to 0.78 and the inter-item correlation ranged from 0.26 to 0.55. A floor effect was found for Relationship Worry for the patients with 20.6% scoring the lowest value. No floor or ceiling effect was found for any of the factors for general practitioners.

### Correlations between derived factors

The correlation between the derived factors was low. For both samples, Closeness correlated negatively with Independence (Patients: r = -0.018; General practitioners: r = -0.264) and negatively with Relation Worry (Patients: r = -0.082; General practitioners: r = -0.169). Relationship Worry correlated positively with Independence for the general practitioners (r = 0.057) and negatively for the patients (r = -0.071).

## Discussion

### Main findings

The Danish version of the RSQ was evaluated on the basis of samples of general practitioners and cancer patients. The data quality was high, with very few missing values and no ceiling or floor effects. The internal consistency and the inter-item correlations were low or moderate for the four subscales. The reliability was acceptable for the Fearful scale, but not for the three other scales. The factor structure could not be confirmed in a CFA. An EFA revealed a similar three-factor structure for both the patients and the general practitioners. These factors were identified as Independence, Relationship Worry, and Closeness in accordance with the included items.

The data quality of the original four subscales was high, however, three items in the patient sample had a higher percentage of missings (items 11, 21, 29) than the other items. These items ask about romantic partners and it is possible that due to the higher age among the patients that more patients were single/widowed and that the missings represent a difficulty in answering these questions. In our translation, we had added in parentheses “current or past” after partners to indicate that even previous partners could be considered when answering the questions but some participants may still have had difficulty in answering these questions. It could also be the case that some participants have never had a romantic partner and were therefore not able to answer these questions. This is a slight concern for the use of the RSQ and it would be optimal if there were directions for participants on how to fill out these questions if they don’t have a current partner or if they have never had a romantic partner. It could be valuable information in the assessment of attachment to know if the participant has never had a romantic partner.

The internal consistency of the original four subscales was low to moderate which indicate that the construct was not fully captured in the original subscales. Surprisingly, the internal consistency of all the 17 items included in the four subscales was slightly better than the internal consistency of the four individual subscales. The internal consistency for the three factors found through the EFA was better than that for the original subscales but still not satisfactory with low average inter-item correlation and a floor effect for the factor Relationship Worry among the patients. One way to improve the validity of the three new factors could be the addition of new items to unfold the constructs.

The EFA we carried out on the 30 items yielded a three-factor structure including 14 items and, hence, excludes 17 of the 30 items. The original four subscales included a total of 17 items and nine of these items were also included in our suggested three-factor model. It is problematic to have a 30 items scale where about half of the items are redundant in the scoring. Before further use of the new scoring, a validation of the three-factors model should be confirmed in other studies with various samples. As with the 17 items used in the scoring of the four subscales it is difficult to determine whether all the 30 items should be used when assessing participants’ attachment or whether the 14 items on their own will yield the same responses from participants compared to the presentation of all 30 items.

The RSQ has been a popular scale especially for use in medical settings due to its general measure of close relationships. However, with problems in confirming its validity another scale such as the Experience in Close Relationship [20] may be of preference.

### Strengths and limitations

The RSQ was translated into Danish using the forward-backward translation procedure, which allows comparison of the Danish version with others studies using the RSQ. When English concepts were used in different items, care was taken to ensure consistent terminology in the translation of the corresponding Danish terms.

The validation of the Danish version was carried out on two large samples. The samples were very different, which gives a broader prospective of how the factorial structure for the RSQ could be. Since the attachment system is activated in times of distress and illness, one would expect the two samples to differ in elicited attachment behavior. Being diagnosed with cancer can bring up strong feelings of anxiety about dependency on others for medical treatment and social support and may also activate desires for closeness, on the one hand, or independence, on the other. The EFA results for both samples were very similar, which indicates that the factorial structure of the RSQ is not affected by situational change. The samples in this validation differ from the sample of undergraduate students in which the RSQ was originally tested [33]. As a consequence of the stressful life circumstances of the cancer patients, we would expect their attachment systems to be more activated than that of undergraduate students. However, this difference between samples parallels the difference between cancer patients and general practitioners and as we found comparable factor structures in these two samples, we would expect the same factor structure to be revealed among students. Future studies have to verify this.

A selection bias may have occurred as patients and general practitioners with a dismissing or fearful type of attachment might have chosen not to complete the RSQ due to their general distrust in others. This could have underestimated the prevalence of these characteristics in both samples.

The test-retest was carried out on 76 general practitioners and was found acceptable for the Fearful scale, but not for the three other scales. It could be argued that the small number of general practitioners in the test-retest population implied that minor divergences led to large fluctuations of the estimated ICC. The test-retest period varied in length, but this is not considered an issue since attachment style is generally regarded as relatively stable over time. The test-retest variability is, consequently, likely to be related to specific major events, which could happen at any time in the general practitioners’ life, rather than a certain length of time. It would have been interesting to see if the patients’ attachment style may prove to be stable over time, or if their illness experience may affect their attachment style, but this was not possible in this study.

### Comparison to other studies

Collins and Read carried out an EFA on the RSQ scale as well and found three factors, *Dependency*, *Anxiety* and *Closeness* and named the scale the Adult Attachment Scale [29]. This scale differs from the RSQ but the three factors they found are somewhat comparable with the factors found in our study. Fontanil, Ezama and Alonso carried out an EFA on the Spanish version of the RSQ scale and found three similar factors *Fear of rejection or abandonment*, *Desire for closeness* and *Preference for independence* [30]. It is noteworthy that some items load on different factors across the studies. This discrepancy may be explained by the number and selection of items included in the analyses of the studies [33]. It is also important to note that the three factors found in ours and in other studies may resemble aspects of attachment, but, even so, cannot be classified as attachment styles.

To our knowledge, no CFA of the RSQ has been able to verify the four attachment subscales proposed by Bartholomew and Horowitz [19]. A plausible explanation may be that the four subscales result from a combination of the two dimensions (model of self and model of other); this implies that each subscale consists of items which may reflect both the model of self and the model of others [33]. A natural next step would be to perform a CFA to document the presence of a model of self and a model of other. However, since both models are derived from equations that include all the items of the subscales (see method section), the possibilities to explore these two dimensions through CFA and EFA seem fairly limited. Initially, Bartholomew attempted to develop coding protocols to measure the dimensions of self and other model directly, but this proved too difficult because of the interdependence of the dimensions [45].

Other studies have found two factors underlying the RSQ scale [34, 46–48]. These factors generally seem to represent the dimensions *Attachment avoidance* and *Attachment anxiety*, which, according to Griffin and Bartholomew are associated with the model of other and the model of self [45]. As no CFA to date has supported the proposed attachment subscales, the RSQ as a psychometrically valid instrument is questioned, and further external and internal validation seem to be required.

## Conclusion

This study provides a Danish version of the RSQ, which has been translated, standardized, and culturally adapted to a Danish setting. The data quality of the scale was generally good, except for low internal consistency. A CFA of the Danish version of the RSQ tested among general practitioners and cancer patients demonstrated a poor fit. An EFA demonstrated a similar three-factor structure for both general practitioners and cancer patients. The internal consistency of the three factors proposed by the EFA was low to moderate. The three factors were validated in a confirmatory factor analyses and had similar attachment themes to the ones found in other studies. Before considering implementation of the three factors identified in this study, replication studies must confirm these findings.
